# Compensatory Cross-Modal Plasticity Persists After Sight Restoration

**DOI:** 10.3389/fnins.2020.00291

**Published:** 2020-05-12

**Authors:** Theresa G. Mowad, Aimee E. Willett, Mani Mahmoudian, Mikhail Lipin, Armin Heinecke, Albert M. Maguire, Jean Bennett, Manzar Ashtari

**Affiliations:** ^1^Department of Ophthalmology, Center for Advanced Retinal and Ocular Therapeutics, University of Pennsylvania, Philadelphia, PA, United States; ^2^The Edward Via College of Osteopathic Medicine, Blacksburg, VA, United States; ^3^Green Clinic, North York, ON, Canada; ^4^Department of Cognitive Neuroscience, Maastricht University, Maastricht, Netherlands; ^5^Department of Ophthalmology, F.M. Kirby Center for Molecular Ophthalmology, Scheie Eye Institute, University of Pennsylvania, Philadelphia, PA, United States; ^6^Center for Cellular and Molecular Therapeutics, The Children’s Hospital of Philadelphia, Philadelphia, PA, United States; ^7^Department of Radiology, University of Pennsylvania, Philadelphia, PA, United States

**Keywords:** low vision, sight restoration, RPE65 gene, cross-modal plasticity, auditory, resting state functional connectivity, functional magnetic resonance imaging

## Abstract

Sensory deprivation prompts extensive structural and functional reorganizations of the cortex resulting in the occupation of space for the lost sense by the intact sensory systems. This process, known as cross-modal plasticity, has been widely studied in individuals with vision or hearing loss. However, little is known on the neuroplastic changes in restoring the deprived sense. Some reports consider the cross-modal functionality maladaptive to the return of the original sense, and others view this as a critical process in maintaining the neurons of the deprived sense active and operational. These controversial views have been challenged in both auditory and vision restoration reports for decades. Recently with the approval of Luxturna as the first retinal gene therapy (GT) drug to reverse blindness, there is a renewed interest for the crucial role of cross-modal plasticity on sight restoration. Employing a battery of task and resting state functional magnetic resonance imaging (rsfMRI), in comparison to a group of sighted controls, we tracked the functional changes in response to auditory and visual stimuli and at rest, in a group of patients with biallelic mutations in the *RPE65* gene (“RPE65 patients”) before and 3 years after GT. While the sighted controls did not present any evidence for auditory cross-modal plasticity, robust responses to the auditory stimuli were found in occipital cortex of the RPE65 patients overlapping visual responses and significantly elevated 3 years after GT. The rsfMRI results showed significant connectivity between the auditory and visual areas for both groups albeit attenuated in patients at baseline but enhanced 3 years after GT. Taken together, these findings demonstrate that (1) RPE65 patients present with an auditory cross-modal component; (2) visual and non-visual responses of the visual cortex are considerably enhanced after vision restoration; and (3) auditory cross-modal functions did not adversely affect the success of vision restitution. We hypothesize that following GT, to meet the demand for the newly established retinal signals, remaining or dormant visual neurons are revived or unmasked for greater participation. These neurons or a subset of these neurons respond to both the visual and non-visual demands and further strengthen connectivity between the auditory and visual cortices.

## Introduction

In the long-term absence of our main sensory system, the brain areas dedicated to these functions may undergo structural and functional changes to host other intact senses. This process is referred to as cross-modal plasticity. To compensate for the loss of a sense, the brain creates or strengthens corticocortical or subcorticocortical connections between the deprived and intact senses (Klinge et al., 2010; Beer et al., 2011; Collignon et al., 2013). The primary visual cortex in low-vision and blind individuals is known to process non-visual information such as auditory signals (Sadato et al., 1996; Roder et al., 1999; Weeks et al., 2000; Bavelier and Neville, 2002; Voss et al., 2004; Gougoux et al., 2005, 2009; Collignon et al., 2007, 2009, 2011a,b, 2013; Ptito et al., 2008; Bedny et al., 2010; Klinge et al., 2010; Frasnelli et al., 2011; Watkins et al., 2013; Kupers and Ptito, 2014; Cecere et al., 2014; Heimler et al., 2014; Renier et al., 2014; Jiang et al., 2016; Dormal et al., 2016) and tactile (Sadato et al., 1996, 2002, 2004; Zangaladze et al., 1999; Burton et al., 2002; Merabet et al., 2004; Pietrini et al., 2004; Goyal et al., 2006; Amedi et al., 2007, 2010; Bonino et al., 2008; Reich et al., 2011; Leo et al., 2012), while also controlling speech comprehension and semantic processing (Roder et al., 2002; Burton, 2003; Noppeney et al., 2003; Raz et al., 2005; Bedny et al., 2011; Pasqualotto and Proulx, 2013). Just as the visual system undergoes cross-modal plasticity to process non-visual senses, other sensory systems, such as the auditory system, can also undergo cross-modal plasticity to process vision (Doucet et al., 2006; Sharma et al., 2007; Kral and Sharma, 2012; Campbell and Sharma, 2014; Cattaneo et al., 2014; Almeida et al., 2015; Stropahl et al., 2015; Dewey and Hartley, 2015; Bola and Borchardt, 2016; Anderson et al., 2017; Glick and Sharma, 2017).

While much of these studies have focused on identifying cross-modal plasticity in the absence of a sensory input, little has been reported about whether these neuroplastic changes of the brain have an adaptive or maladaptive effect on restoration of the deprived sense. On the one hand, cross-modal plasticity is reported as an adaptive process for the enhancement of the remaining senses in blind or deaf individuals. On the other hand, this process has been considered maladaptive for the optimal success of reinstating the original sense (vision or auditory, reviewed by Heimler et al., 2014). The maladaptive aspect of the cross-modal plasticity has largely transpired from studies on restoration of the auditory function [through cochlear implant (CI)] rather than studies on visual recovery. Although CIs have technologically advanced over time and currently comprise the most widespread and successful neuroprosthesis available (Wilson and Dorman, 2008; Kral et al., 2019), patients with early or late onset of deafness have presented with mixed responses to this device. Among factors such as age at onset (prelingual or postlingual), duration of deafness, and implantation age, cortical changes of the auditory cortex through takeover by other senses (cross-modal plasticity), in particular vision, have been considered as one factor for the degree of the patients’ response to the CI (Doucet et al., 2006; Buckley and Tobey, 2011; Sandmann et al., 2012; reviewed Collignon et al., 2011a; Kral and Sharma, 2012; Voss, 2013; Sharma et al., 2015; Kral et al., 2019). The maladaptive aspects of the cross-modal plasticity within the auditory cortex for the CI have been mainly attributed to positive correlations between the higher levels of cortical reorganization and poor CI outcomes (Giraud et al., 2001; Oh et al., 2003; Green et al., 2005; Viola et al., 2011; Sandmann et al., 2012). However, such correlations should not be considered a direct cause for patients’ poor response to CIs by the auditory cortex occupation of the visual functions (Lyness et al., 2013) and the assumption that success of CI is critically dependent on reducing the auditory cortex activations by visual language (Rouger et al., 2012). A support to this argument comes from an elegant study by Strelnikov et al. (2013) that specifically focused on assessing the correlations between brain activations shortly before implantation with the level of auditory recovery 6 months post CI in a group of post-lingual deaf subjects. Using a speech-processing task, authors reported the highest positive correlations with areas involved in visual processing such as the occipital cortex and the posterior temporal cortex known for audiovisual integration (Strelnikov et al., 2013), indicating a strong link between the visual modalities’ functional strength and the proficiency level of auditory recovery. Authors concluded that synergy between the auditory and visual areas is a crucial factor for cross modal-plasticity and speech comprehension recovery in postlingual CI subjects (Strelnikov et al., 2013). Also a recent longitudinal study (Anderson et al., 2017) assessed the effect of cross-modal visual speech and the success of CI in a group of 17 adults with bilateral profound deafness before and 6 months following CI use. Contrary to previous findings, authors reported increased cross-modal activation of auditory brain regions by visual speech from before to after implantation, which was associated with better speech understanding, and both auditory and speech functions developed in synchrony after cochlear implantation (Anderson et al., 2017). The concluding remarks from this report rejected the previous concept on the maladaptive effects of cross-modal plasticity by visual speech. In fact, the authors suggested that cross-modal plasticity may have indeed played a positive adaptive role in restoring patients’ auditory functions (Anderson et al., 2017). In another study, Chen et al. (2017), using functional near infrared spectroscopy, showed that increase in cross-modal functional connectivity after the CI was associated with better speech recognition abilities, pointing to a new pattern of functional reorganization that is related to successful hearing restoration with a CI. It is important to note that majority of patients studied in the aforementioned reports were postlingually deaf and not from congenitally deaf individuals. However, a recent study on the higher-order auditory cortex in a group of cats with congenital deafness (Land et al., 2016), using brain mapping with microelectrode array, presented supporting evidence that visual cross-modal responsiveness did not interfere with the success of hearing restoration through CIs (Land et al., 2016).

Unlike the CI reports, studies on vision restorations and the fate of cross-modal components are scarce, however, the role of cross-modal plasticity for vision restoration in patients with low vision or complete blindness faced similar opposing arguments. On the one hand, the takeover of the visual cortex by other senses such as auditory or tactile functions has been considered to have enormous adaptive advantage in the everyday lives of visually deprived individuals (Saenz et al., 2008; Roder et al., 2013). On the other hand, such cortical reorganizations have been anticipated to play a maladaptive role for possible sight restoration techniques, such as retinal prosthetic devices, stem cell transplantation gene therapy (GT), cataract removal surgery, and so on, particularly for older patients who have passed the critical window for vision development (Legge and Chung, 2016). While a range of sensory functions including auditory can be altered as a result of blindness or low vision, because of close interactions between vision and hearing, this report will focus primarily on the impact of vision restoration on auditory cross-modal plasticity. One of the earlier studies demonstrating the persistence of auditory cross-modal function after vision reversal was reported by Saenz et al. (2008). This study reported auditory motion responses within the MT/V5 area, known to be dedicated to visual motions (cross-modal), in two subjects (MM and MS) who had been blind since childhood and regained partial vision in adulthood. Normal-sighted controls performing the same task did not show similar auditory responses (Saenz et al., 2008). Authors concluded that auditory and visual responses coexist after sight recovery even after a long period of blindness. This study also concluded that colonization of the MT area by the motion sensitive auditory stimuli may indeed have played a critical role in preserving the neuronal connectivity of the MT area to in turn respond to visual stimulation. In another study, Roder et al. (2013) employed electro-encephalograms to study selectivity of responses to faces compared to objects in a group of individuals who were born with congenital cataracts. These individuals then regained partial vision through bilateral cataract removal surgery at different ages (Roder et al., 2013). The authors demonstrated that despite a long period of blindness (14 years) and the natural formation of the cross-modal functions as a consequence of such a long period of visual deprivation, visual brain areas were successfully recruited for visual processing. The study reported that such brain behavior may be due to partial reversal of a cross-modal component. Alternatively, the formation of cross-modal plasticity may have in fact contributed to the maintenance of the visual neurons (Roder et al., 2013). A separate report by Dormal et al. (2015) studied a single subject (KL) before and 1.5 and 7 months after vision restoration. The authors reported an increase in auditory activations overlapping the visual responses in the primary visual areas 7 months after surgery. However, these auditory cross-modal responses were reported to be decreased in the extrastriate occipital regions after surgery. Overall, the study concluded that the primary visual cortex maintained its involvement in the processing of non-visual information despite sight restoration (Dormal et al., 2015). A recent report by Jiang et al. (2016) compared an individual subject (MM) who acquired vision at the age of 46 years after becoming blind at age 3 years with a group of early and late blind as well as a group of sighted controls to study auditory motion processing. The authors reported enhanced auditory motion responses in the MT area and reduced functionality in the right planum temporale among blind subjects and the sight-recovered individual. The study concluded that the cortical plasticity that occurs as a result of early blindness is permanent and is not reversed even after vision is restored (Jiang et al., 2016). The persistence of the auditory cross-modal component after vision recovery was also supported in a study that compared a group of six individuals who had been born blind due to dense bilateral cataracts and regained sight when they were treated at 5–24 months of age to a group of normally sighted participants and an additional group of individuals who had had pattern vision in childhood but later developed visual impairments (Guerreiro et al., 2016). The study reported that cataract-reversal individuals showed significant auditory motion responses as compared to the other two control groups. These results confirm the previous findings reported for the persisting cross-modal reorganization of MT/V5 area in sight-recovered individuals (Saenz et al., 2008; Jiang et al., 2016).

Recently, a successful clinical trial at the Children’s Hospital of Philadelphia (CHOP) and University of Pennsylvania (UPenn) on low-vision patients has led to Luxturna, the first US Food and Drug Administration (FDA)–approved retinal GT drug. Luxturna aims to reverse blindness in patients with Leber congenital amaurosis (LCA) caused by biallelic *RPE65* mutations. Leber congenital amaurosis is a rare ocular disease characterized by severe visual impairments from birth or early infancy, night blindness, poor or absent pupillary light responses, mild to severe nystagmus, and abnormal electroretinogram (Kumaran et al., 2017). The *RPE65* gene, which encodes the retinal pigment epithelium-specific 65-kDa protein, is the most common form of LCA, called LCA type 2 (Redmond et al., 1998; Jin et al., 2005; den Hollander et al., 2008). Because of its favorable properties with respect to slow degenerative process (and thus presence of treatable cells through childhood) and reports of efficacy in animal models, RPE65 patients (LCA2) have been subject to the most clinical trials^1^. Further details on the CHOP/Penn RPE65 clinical trials are provided in section Materials and Methods.

With the approval of Luxturna, there is renewed interest in studying the crucial role cross-modal plasticity may play on sight restoration. Having access to a group of patients before and after receiving retinal GT provides a unique opportunity to shed light on the highly debated controversial views that have been challenged in both the auditory and vision restoration reports for decades. The main goals of this study were threefold. First, we aimed to evaluate whether RPE65 patients present with auditory cross-modal plasticity. Second, we aimed to investigate the fate of cross-modal plasticity when these patients undergo retinal GT and regain their vision. Lastly, we aimed to identify the adaptive or maladaptive role of cross-modal plasticity in sight restoration. Based on the previous literature regarding cross-modal plasticity and vision restoration, we hypothesize that RPE65 patients who partially regained their vision through retinal GT would preserve their cross-modal plasticity long after their vision reversal. To evaluate our hypothesis, we assessed cortical activations in response to visual and auditory stimulation, as well as in a group of RPE65 patients before and 3 years after their second (contralateral eye) retinal GT to compare with a group of demographically matched sighted controls. Furthermore, resting state functional magnetic resonance imaging fMRI (rsfMRI) data were collected for both groups to compare the functional connectivity of the primary auditory and visual areas using the primary visual (BA17) and the primary auditory (BA41) areas as anatomical seed points.

## Materials and Methods

### GT Clinical Trials

Leber congenital amaurosis is a rare ocular disease, usually inherited in an autosomal recessive fashion. Leber congenital amaurosis has been associated with at least 20 different genetic mutations (Bowne et al., 2011). The gene encoding retinal pigment epithelium-specific protein 65 kDa (RPE65) is involved in one of the more common forms of LCA called LCA type 2 (LCA2) (Redmond et al., 1998, 2005; Jin et al., 2005; Moiseyev et al., 2005). One of the first presentations of LCA that may occur in infancy is the lack of visual responsiveness and fast eye movement known as roving eye or nystagmus. The disease is most prominent in dim or no light environment (night). While patients may see in bright light early on in their lives, due to degenerative nature of the disease, their visual functions worsen over time. RPE65 patients (LCA2) are good candidates for gene transfer therapy as the degeneration of retinal cells is relatively slow. There are several clinical trials that have carried out GT for individuals with *RPE65*-mediated disease^1^. The CHOP/UPenn is the first GT program to carry out a clinical trial where pediatric and adult subjects with mutations in the *RPE65* gene received GT to their worse-seeing eye (Clinical Trial no. NCT00516477). According to the results from the first clinical assessment of these patients, published shortly after receiving unilateral subretinal injection in one eye (Maguire et al., 2008), each patient had a modest improvement in measures of retinal function on subjective clinical tests such as visual acuity and visual fields (Maguire et al., 2008). In 2011, the same subjects underwent retinal GT in their contralateral eye in a follow-on (FO) study of phase 1 (Clinical Trial no. NCT01208389) (Bennett et al., 2012). After injection, results from light sensitivity (red, blue and white), pupillometry, and other clinical tests, as well as fMRI, showed that each of these “second” eyes became far more sensitive even though they had been severely impaired for more than 2.5 decades (and more than 4.5 decades in one individual) (Bennett et al., 2012). Clinical testing and brain imaging results consistently showed favorable response to the retinal GT, particularly after receiving FO subretinal injection in their contralateral eye. A 3-year clinical follow-up of these patients showed GT to the contralateral eye to have strong and stable visual improvement (Bennett et al., 2016). Parallel to the clinical results, a 3-year follow-up fMRI results provided a complementary information on these patients (Ashtari et al., 2017), attesting to the success and durability of the retinal GT. Although, retinal GT did not bring the degree of visual functions to the level of normal-sighted individual, the significance in visual augmentation in RPE65 patients and its continuous success for this clinical trial and the final phase 3 clinical trial eventually resulted in receiving FDA approval for this revolutionized therapy in December of 2018.

### Study Participants

A subset of FO patients (8/12) participated in a separate neuroimaging study. Additionally, eight demographically matched normal-sighted controls were recruited. As shown in Table 1, all RPE65 patients received their FO subretinal injection in the left eye, except for one patient who received the FO treatment in the right eye (Bennett et al., 2016). Subjects with a current or past psychiatric diagnosis, history of alcohol or drug abuse, known neurological disorders, history of head injury, or current use of psychotropic medications were excluded from the study. All study participants provided written informed consent (if 18 years or older) or written assent and parental permission (if younger than 18 years). This study was Health Insurance Portability and Accountability Act of 1996 compliant and approved by the internal review board at CHOP and UPenn. RPE65 patients and controls did not significantly differ in age, gender, and handedness (Table 2).

**TABLE 1:** RPE65 patient demographics.

Subject ID	Age (years) at readministration and baseline fMRI	Readministered Eye	*RPE65* mutation(s)
			
NP01	30	Left	E102K/E102K
NP02	30	Left	E102K/E102K
CH08	12	Left	F530fs/F530fs
CH09	11	Right	R124X/K297del1aggA
CH10	14	Left	IVS1 + 5g > a/F530del1ttc
CH11	27	Left	V473D/V473D
CH12	46	Left	K303X/W431C
NP15	14	Left	D167W/H313R

**TABLE 2:** Statistical comparison of the RPE65 patients and sighted controls.

	Controls	Patients	*p*
*n*	8	8	
Age at baseline			
Mean (SD)	23.3 (12.0)	23.0 (12.4)	0.97*
Range	(11.0–44.0)	(11.0–46.0)	
Gender			1.00^†^
Female	2 (25.0%)	3 (37.5%)	
Male	6 (75.0%)	5 (62.5%)	
Race			1.00^†^
Caucasian	7 (87.5%)	7 (87.5%)	
Indian	0 (0.0%)	1 (12.5%)	
More than one race	1 (12.5%)	0 (0.0%)	
Handedness			1.00^†^
Left	0 (0.0%)	1 (12.5%)	
Right	8 (100.0%)	7 (87.5%)	

*Equal variance t-test. ^†^Fisher exact.

### Neuroimaging Protocol

All fMRI experiments were carried out at CHOP on a research dedicated 3T Siemens Verio system (Siemens Campus Erlangen, Erlangen, Germany) using a 32-channel head coil. A single operator carried out all scans, and each subject’s head motions were monitored using the Siemens’ proprietary real-time fMRI monitoring software (Ashtari et al., 2014). Prior to and 3 years after the FO clinical trial, all RPE65 patients underwent auditory, visual, and rsfMRI using the blood oxygenation level–dependent (BOLD) imaging sequences and three-dimensional (3D) structural MRI. Sighted controls underwent the same imaging protocol at baseline. For both the auditory and rsfMRI acquisitions, all light sources were turned off or powered down to eliminate other possible sources of visual stimulation.

#### Auditory and Visual fMRI Acquisitions

The auditory and visual task data were acquired in axial orientation parallel to the anterior–posterior commissure (AC-PC) plane. The following sequence parameters were employed: 3,000 ms time to repetition (TR), 30-ms echo time (TE), 90° flip angle (FA), 3 mm slice thickness, 64 × 64 matrix size, and 46 number of slices with a total acquisition time of 3:12 min for auditory and 4:33 min for the visual tasks. The auditory and visual tasks consisted of 62 and 90 brain volumes, respectively. Three brain volumes, used to reach T1 equilibrium, at the beginning of each fMRI task were discarded. A transistor–transistor logic pulse was used to automatically start the stimuli in sync with the start of fMRI acquisition (Ashtari et al., 2011; Bennett et al., 2012).

#### rsfMRI Acquisition

Resting state fMRI is characterized by the low-frequency BOLD signal components that significantly correlate within or between multiple remote brain regions, forming specific functional connectivity networks. Resting state is acquired while the subject is lying inside the magnet with no sensory stimulation applied (Biswal et al., 1995; Lowe et al., 1998). In addition to the visual and auditory paradigms, all participants underwent an rsfMRI experiment with the same acquisition parameters as the task-based fMRI paradigms. A total of 91 brain volumes were acquired in the axial orientation parallel to the AC-PC plane with a total acquisition time of 4:33 min. Subjects were instructed to relax while remaining still. Similar to the auditory task, all other light sources were turned off to eliminate confounding visual stimulation.

#### 3D Anatomical Acquisition

T1-weighted images of the whole brain were obtained using the 32-channel head coil at 0.8 mm isotropic resolution using the 3D magnetization prepared rapid acquisition gradient echo sequence with the following sequence parameters: 1,200 ms inversion time; 2,080 ms TR; 2.54 ms TE; 90° FA; 192 slices; 320 × 320 matrix; and a total acquisition time of 7:04 min.

### Auditory Paradigm

**FIGURE 1:**
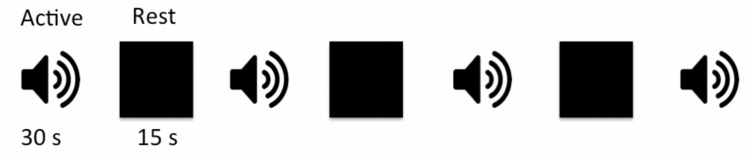
Schematic block diagram of the auditory paradigm. The auditory task consisted of four active sound blocks interspersed with three 15-s rest blocks. Each sound block was 30 s long containing 10, 2,000 ms long sound pairs with 1,000 ms between the two sound pairs to allow for subject response. There were a total of 40 randomly selected sound pairs, from a list of 72 wave forms, presented in each experiment.

Subjects were presented with pairs of sounds and asked to press a button if sounds were the same and not respond if they were different. Sound stimuli were randomly selected from a library of 72 sound pairs that were generated in-house using MATLAB program (MathWorks, Natick, MA, United States). Each sound in a pair consisted of two sinusoids with a frequency range of 500–1,800 Hz, and a mean frequency of 1,174 ± 300 Hz and 1,253 ± 327 Hz for the first and second sound, respectively. The duration for one sound pair discrimination epoch including the time for subject response was 2,000 ms with a 1,000 ms interval between consecutive sound pairs. Each active block contained 10 sound pairs and lasted 30 s. The auditory paradigm consisted of a total of four sound blocks interleaved with three 15 s rest blocks for total task duration of 4:12 min (Figure 1). A 3 s instructional block appeared at the beginning to instruct the participants and account for spin equilibrium. No auditory stimuli were presented during the rest blocks other than the background scanner noise. Subjects were asked to relax and close their eyes during the whole experiment period. Stimuli were presented through a desktop computer and an MRI-compatible audiovisual system (Northridge CA, United States)^2^. Special silicone gel cushions adhered to the headset attenuated ambient noise by ∼30 dB. The headset was specially designed with suspended ceramic transducer enhancing delivery of a full audio frequency response to the subject. The suspension mechanism prevented the transducer’s membrane to resonate with RF or gradient pulses as opposed to piezo technology that is applied to hard material such as brass. Task programming was carried out using E-Prime (Pittsburgh, PA, United States)^3^.

### Visual Paradigm

**FIGURE 2:**
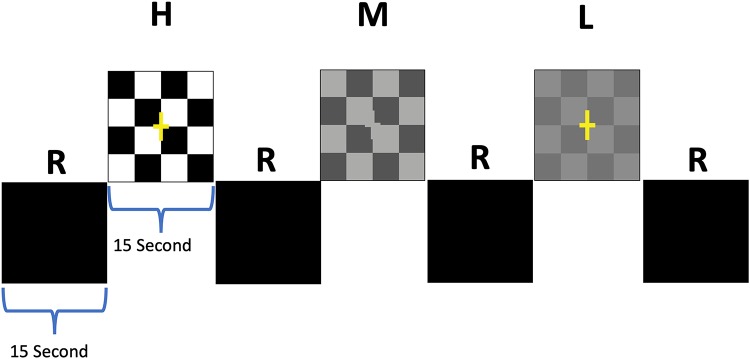
Schematic block diagram of the visual stimuli. The checkerboard paradigm consisted of 15 s active blocks of contrast-reversing (8 Hz) checkerboards interleaved with 15 s presentation of a blank screen as rest blocks (R). Active blocks were comprised of 3 blocks for each contrast that were randomly presented for high (H), medium (M), and low (L) contrasts interspersed with 9 rest blocks (Ashtari et al., 2011).

The visual paradigm consisted of 15 s active blocks of contrast-reversing (8 Hz) checkerboards with low-, medium-, and high-contrast checkers interleaved with 15 s of blank, black screens as control blocks (Figures 2A,B; Ashtari et al., 2011). Additionally, subjects were asked to press a button as soon as they could detect checkerboard patterns. Each participant completed two runs with stimuli presentation limited to either the left or the right eye. Single eye stimulation was performed electronically via the VisuStim goggle system (Resonance Technology Inc., Northridge, CA, United States), allowing the stimuli to be presented to each eye separately. Stimuli were presented through a desktop computer and an MRI-compatible audiovisual system. Task programming was carried out using E-Prime.

### Preprocessing of Functional Data

All fMRI data were processed using Brain Voyager 21.4 (BV21.4; Maastricht, the Netherlands)^4^. Preprocessing of data included slice scan time correction, 3D motion correction, high-pass temporal filtering, and spatial smoothing. Sinc interpolation was used for scan time correction to ensure that all voxels in the volume represented the signal simultaneously. A high-pass temporal filter with a cutoff frequency of two cycles per run was applied to remove a drift. Spatial smoothing was performed using a 4 mm full-width at half-maximum Gaussian filter. In addition to monitoring the subjects’ motions in real time, additional uncorrected subject motions were corrected using an algorithm implemented in BV21.4, calculating six translational and rotational motion parameters in relation to the first acquired volume using sinc interpolation.

### Preprocessing of Resting State Data

In addition to the aforementioned typical data preprocessing, rsfMRI time series required additional preprocessing to exclude coherent fluctuations that are unrelated to the neural processes of interest. Removal of cardiac and respiratory confounds was performed by obtaining regions of interest (ROIs) for the white matter and ventricular volumes using the BV21.4 software segmentation algorithm. The final residual resting state time series excluded subject motion, breathing, and cardiac function; this was obtained by the regression of the time courses attributed to these physiological confounds from the original rsfMRI time course.

## Preprocessing of the Anatomical Data

All anatomical data were preprocessed using BV21.4 with the processing steps of intensity inhomogeneity correction, resampling from 0.8 to 1 mm isovoxel and spatial transformation to AC-PC and Talairach (TAL) space. The final TAL transferred brains were extracted from surrounding head tissue and segmented to define the gray matter/WM boundary to reconstruct a folded surface representation for the left and right hemisphere (Kriegeskorte and Goebel, 2001). The individual cortex meshes were used to represent a 3D projection of the functional data time course, as well as performing a more accurate cortex-based group analysis (Goebel et al., 2006).

### Cortex-Based Alignment Group Registration

To improve the spatial correspondence mapping between subjects’ brains beyond TAL space matching, cortex-based alignment (CBA) was performed to align the reconstructed folded meshes, as implemented by BV21.4 (Fischl et al., 1999). This method matches gyri and sulci locations across the brains of each individual subject in order to bring major cortical landmarks into alignment beyond standard normalization. The time course of functional data in volume space for each subject is then attached to the corresponding vertices of the aligned folded cortex resulting in a mesh time course (MTC) for each run of each subject’s functional data. Furthermore, the CBA transformation matrix allows for a more precise alignment of subject specific ROIs, or Brodmann areas, such as the primary auditory (BA41) and primary visual (BA17) cortical labels. This step was performed for a more reliable group-level rsfMRI analyses.

### fMRI Statistical Analysis

Using BV 21.4 for each functional run of an individual subject, a protocol file (PRT) was created representing the duration and timing of each condition for the auditory and visual tasks. A design matrix was then constructed using the defined PRT and data dispersion due to hemodynamic response with a double-gamma function (Friston et al., 1998). Task-based auditory and visual paradigms for each subject and separate runs were then analyzed using the general linear model (GLM), which is mathematically similar to a multiple regression analysis and is based on a univariate statistic for each voxel of the acquired brain volumes. The auditory experiment was analyzed by contrasting the active blocks of sounds with silent blocks (rest periods); the visual task was analyzed by contrasting only high-contrast checkerboard blocks with the rest blocks. To account for the multiple-comparisons problem caused by the thousands of *t*-tests (one per voxel) performed per slice for each brain volume, we used the false discovery rate (FDR) approach (Genovese et al., 2002). Given a desired FDR, the algorithm calculated a single-voxel threshold, which ensured that the voxels beyond that preset threshold contained no more than the specified proportion of false positives approximately. A conservative FDR threshold of at least 5% (*q* = 0.05) was chosen for all fMRI analyses. The corresponding *p*-values reported for each subject and condition were automatically calculated by BV21.4. The multiple comparison problem was further controlled by restricting the final results to a greater than 100 contiguous voxels that individually met the GLM contrast and FDR criteria. Using the cortically aligned MTC data as input, the fixed-effects (FFX) and random-effects (RFX) GLMs at the group level were computed in a similar fashion for all functional data.

### rsfMRI Statistical Analysis

There are two widely used approaches for analyzing an rsfMRI dataset: seed-based ROI correlation or independent component analysis (Calhoun et al., 2003; Weissenbacher et al., 2009). Based on our “*a priori*” hypotheses of assessing functional connectivity between the two brain regions of the primary auditory and primary visual cortices, rsfMRI were analyzed using region-based correlation analysis. This was accomplished by choosing ROIs common across subjects from atlas-derived anatomical locations such as Brodmann atlas positions of BA41 and BA17 as the anatomical seed ROIs for the primary auditory and vision, respectively. Using these seed regions and the residual rsfMRI data, a corresponding time series of each seed network and the whole brain was obtained. Prior to performing group comparison, each subject’s resting state data were then transferred from the time space into a different domain as recommended by Iraji et al. (2016) to ensure each value at a specific time point represents the same effect across subjects. This step was accomplished by normalizing the extracted time course for each region using the GLM model and *z*-transformation as implemented in BV 21.4. The new normalized time courses were then stored as new region-specific design matrix as input to the second-level multisubject FFX and RFX GLM analyses. In principle, this analysis can be performed in both volume and surface levels. However, for a more accurate mapping, the region-specific normalized time courses were obtained in the surface space using the information obtained from CBA for an improved anatomical mapping.

## Results

### Auditory Cross-Modal Plasticity in RPE65 Patients

**FIGURE 3:**
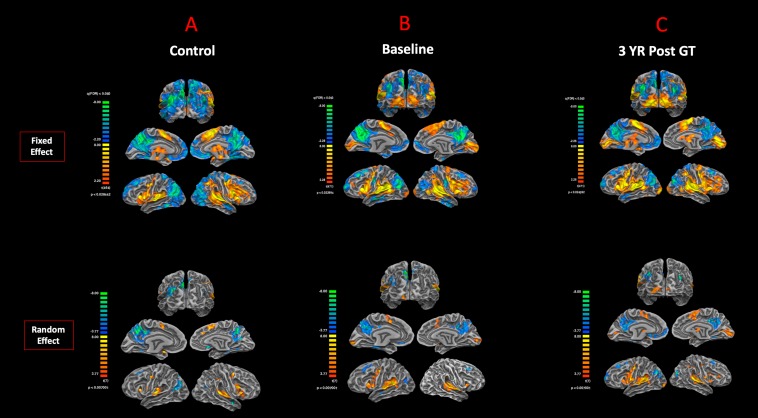
Functional magnetic resonance imaging response to auditory stimulation. The results from the task-based auditory fMRI are presented for **(A)** sighted controls, **(B)** RPE65 patients before gene therapy, and **(C)** RPE65 patients 3 years after GT. The FX effect group analyses were processed at FDR corrected *p* < 0.02 (*q* < 0.05), with an extent threshold of >100 mm^2^, and the RFX analyses were performed at an uncorrected *p* < 0.007 with an extent threshold >100 mm^2^. Sighted controls showed the expected cortical activations in and around the bilateral Heschl’s gyrus, the inferior frontal gyrus (IFG), and the somatosensory cortex and deactivations in the visual cortex and within the default mode network. Compared to sighted controls, baseline RPE65 patients had similar patterns of auditory cortical activation, yet significant cortical activations within the bilateral visual cortices. Three years after retinal gene therapy, RPE65 patients showed the similar cortical activation patterns that were significantly enhanced.

**FIGURE 4:**
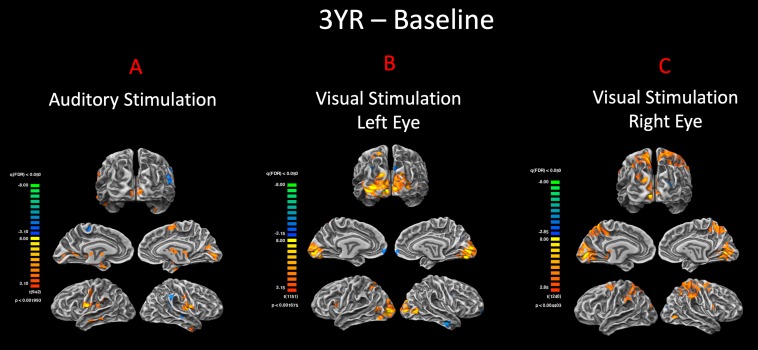
Pre– and post–gene therapy comparison for auditory and visual stimulations. Direct group comparisons of pre– and 3 years post–retinal gene therapy were performed using the FX group-level analysis in response to **(A)** auditory and **(B,C)** visual stimulations. Group results are presented at an FDR-corrected *p* < 0.002 and *p* < 0.005 for auditory and vision, respectively (*q* < 0.05). In response to the same auditory stimulation, RPE65 patients showed increased bilateral auditory activation within the auditory cortex, as well as significant level of cross-modal activations along the bilateral calcarine sulci for their 3-year time point. The group comparison of the visual stimulation results between the baseline and 3 years post–gene therapy time points showed significantly more activations in and around the visual cortex for both the left and right eyes (left > right) 3 years after retinal gene therapy.

The cortex-based fixed-effects (FFX) and random-effects (RFX) group GLM analyses for overall auditory stimulation vs. rest conditions in sighted controls showed activity within and around Heschl’s gyrus, bilateral superior temporal sulcus, sensorimotor cortex, and inferior frontal gyrus, at FDR-corrected *p* < 0.02 for FFX effect (Figure 3A). At an uncorrected *p* < 0.007 for RFX, greater attenuated activation levels in and around Heschl’s gyrus were observed (Figure 3A). Group analyses for sighted controls did not reveal cortical activations of the occipital cortex; however, single-subject analysis shown in Supplementary Figure S1 showed slight activity in the occipital cortex of only one sighted control. As shown in Figure 3A, particularly in the FFX effect group analysis, sighted controls showed expected areas of cortical deactivations in the visual cortex and within the default mode network. In response to the same auditory stimuli and statistical threshold for both FFX and RFX group analyses, the RPE65 patients showed similar patterns of auditory cortical activation to those observed in sighted controls, with the exception of the significant cortical activations in both the left and right primary visual cortices (Figure 3B). Similar default mode network deactivations for BA31 and BA10 areas were observed for RPE65 patients (Figure 3B). Compared to baseline, FFX effect analyses at the same group statistical threshold showed more enhanced and widespread cortical activations in and around Heschl’s gyrus, as well as the bilateral occipital cortices in RPE65 patients 3 years after FO GT (Figures 3B,C). The RFX group analysis confirmed similar results for areas of activations albeit with attenuated magnitude. Compensatory areas of activation were primarily distributed along the bilateral calcarine sulci and occipital poles, and the magnitude of cortical activations for these areas for both the FFX and RFX analyses is quantified to further demonstrate the differences between the levels of auditory cross-modal component before and 3 years post–retinal GT (Table 3). RPE65 patients consistently presented with the expected cortical deactivation in the default mode areas. As shown in Supplementary Figures S2 and S3, single-subject analysis for both baseline and 3 years after FO GT of RPE65 participants showed that all patients, except for CH12, presented with visual cortex activations while performing the auditory fMRI task experiments. To test the significance of the enhanced cortical activations, observed at 3-year FO GT, particularly in the visual cortex, direct group comparisons were performed between baseline and FO auditory time courses using group-level analyses. Significant bilateral visual cortex activations at FDR corrected *p* < 0.002 for FFX effect analysis attesting to the presence of significant compensatory auditory cross-modal activation 3 years after FO GT (Figure 4A).

**TABLE 3:** Quantification of the auditory induced visual cortex activations (auditory cross-modal plasticity presented in Figure 3), at baseline and 3 years post–retinal gene therapy for both the fixed- and random-effects analyses.

Auditory fMRI task results (Figure 3)	FFX* (LH) mm^2^	FFX (RH) mm^2^	RFX^†^ (LH) mm^2^	RFX (RH) mm^2^
Baseline	2,938	3,236	197	648
3 years	3,235	4,180	516	1,039

*FFX = fixed effect. ^†^RFX = random effect. LH, left hemisphere; RH, right hemisphere.

### Visual Functions in RPE65 Patients 3 Years After Retinal GT

**FIGURE 5:**
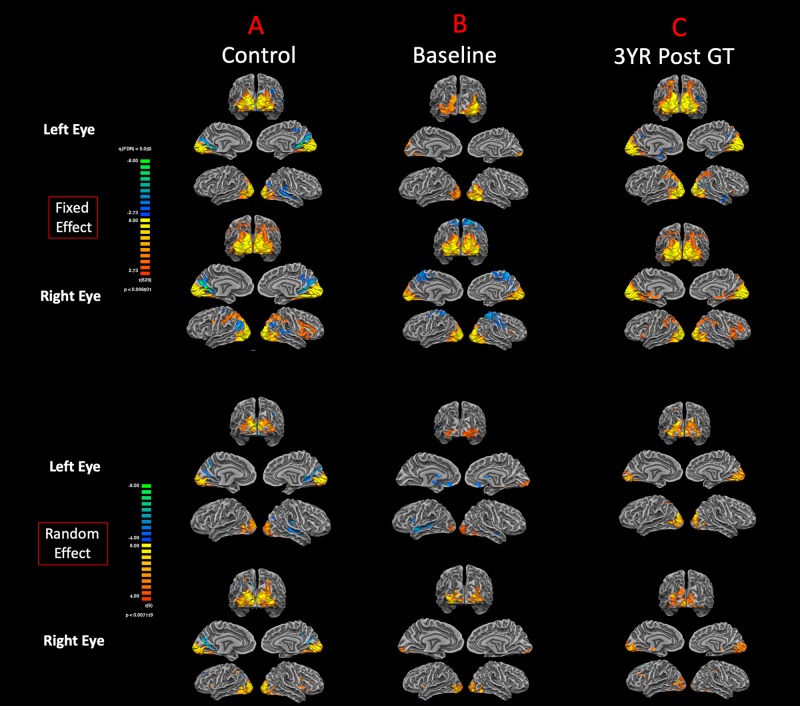
Functional magnetic resonance imaging response to visual stimulation. The visual stimulation tasks were presented either to the left or right eye of **(A)** normal-sighted controls, **(B)** RPE65 patients before GT, and **(C)** RPE65 patients 3 years after GT. All task-based cortical activations were processed using FX analyses at FDR corrected *p* < 0.007 (*q* < 0.05), with an extent threshold of >100 mm^2^ and RFX analyses at an uncorrected *p* < 0.007 with an extent threshold > 100 mm^2^. Sighted controls showed significant bilateral cortical activations in the visual cortex. At baseline, RPE65 patients expressed asymmetric and attenuated hemispheric activations in the left eye because the left eye was untreated in seven of eight patients. However, visual stimulation of the right eye in RPE65 patients displayed nearly symmetrical bilateral activation of the visual cortex. Three years post–FO clinical trial when RPE65 patients’ contralateral eye was treated, symmetrical bilateral visual cortex activations were observed for both left and right eyes.

In addition to the auditory stimulations, visual stimulation experiments were performed to assess visual functions for both the left and right eyes of sighted controls, RPE65 patients at baseline, and 3 years after receiving retinal GT. The cortex-based FFX and RFX effect group GLM analyses for the right and left visual stimulation for high-contrast checkerboard vs. rest conditions showed symmetrical bilateral activations in the primary visual areas at FDR corrected *p* < 0.007 for FFX effect and with diminished activation levels for RFX at an uncorrected *p* < 0.007 (Figure 5A). The sighted control group presented cortical deactivations in the right hemisphere around the auditory cortex and bilaterally within the parieto-occipital sulci (Figure 5A). Group analyses for the left eye visual stimulation of the RPE65 patients at baseline (untreated in 7/8 patients) depicted an asymmetric cortical activation within the visual cortex (Figure 5B). The FFX and RFX effect group results for the right eye stimulation of the RPE65 patients at baseline showed nearly symmetrical bilateral activation of the visual cortex (Figure 5B). The FFX and RFX group results for the same RPE65 patients at 3-year time point after retinal GT, in response to the same visual stimulation protocol, showed significantly enhanced symmetrical bilateral visual cortex activations for both left and right eyes (Figure 5C). The cortical deactivation areas observed for sighted controls in the auditory and the parieto-occipital sulci were not observed in either FFX or RFX effect group analyses of the RPE65 patients at baseline or 3-year FO analyses (Figures 5B,C).

To demonstrate the significance of the enhanced visual functions observed at 3-year FO time point, direct group comparisons were performed between the visual responses at the two time points. The FFX effect group analyses revealed significant bilateral visual cortex activations at FDR corrected *p* < 0.004 and *p* < 0.002 for the right and left eyes, respectively. These results are consistent with our earlier reports on the efficacy of the FO GT treatment (Bennett et al., 2016; Ashtari et al., 2017) and further confirm the presence of significant visual functions in both the right and left eyes of the RPE65 patients long after receiving retinal intervention.

### Connectivity of the Primary Auditory Areas (BA41) to the Visual Cortex

**FIGURE 6:**
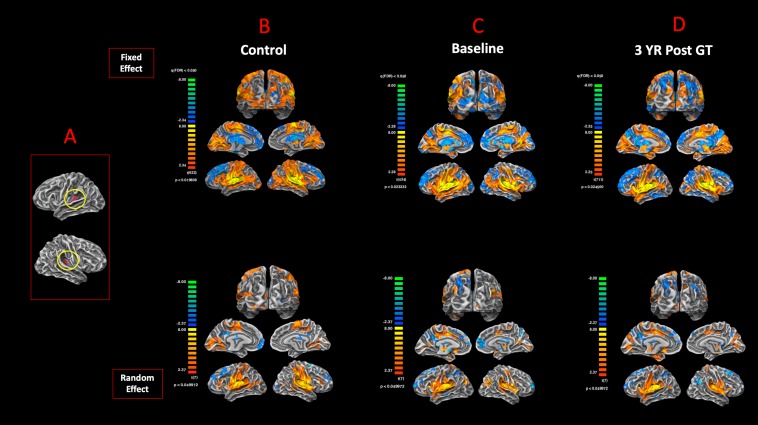
Functional connectivity of the primary auditory areas. Anatomical locations for both the left and right Brodmann primary auditory areas (BA41) **(A)**. The functional connectivity group results for FX and RFX analysis are presented for **(B)** sighted controls, **(C)** RPE65 patients at baseline, and **(D)** RPE65 patients 3 years post–gene therapy. All fixed-effects group analyses were performed at FDR corrected *p* < 0.02 (*q* < 0.05), with an extent threshold of >100 mm^2^, and all RFX analyses were performed at an uncorrected *p* < 0.05 with an extent threshold >100 mm^2^. Sighted controls showed significant bilateral functional connectivity between auditory, sensorimotor, frontal, and visual cortices. At baseline, compared to controls and post–gene therapy time point, RPE65 patients expressed similar, yet attenuated connectivity patterns particularly along the bilateral visual cortices. Three years after receiving their gene therapy, RPE65 patients’ connectivity of the primary auditory areas to the visual cortex significantly increased, yet remained attenuated as compared to the observed connectivity of the sighted controls.

The surface representation of the functional connectivity of the auditory cortex to other brain regions using BA41 is presented in Figure 6. Locations for the left and right Brodmann primary auditory areas (BA41) as anatomical seed points are depicted in Figure 6A. Sighted controls presented with significant connectivity between the BA41 seed and the rest of the brain, including the bilateral primary visual areas along the calcarine and parietal-occipital cortices, particularly for the fixed-effects analysis, although much of this connectivity pattern is preserved in the RFX analyses (Figure 6B). Sighted controls also presented with negatively active areas involving the default mode. Similar to the sighted controls, RPE65 patients, for both baseline and 3 years after FO GT, presented highly significant connectivity between the BA41 seed areas and other brain regions, particularly the bilateral calcarine cortex and parietal-occipital areas (Figures 6C,D). Comparing RPE65 patients at baseline to 3-year FO after GT, much more enhanced connectivity pattern is observed for the 3-year time point along the calcarine sulci and within the parieto-occipital areas. Table 4 shows the quantified areas of functional connectivity activations along these areas for both the FFX and RFX analyses before and 3 years after retinal GT.

**TABLE 4:** Quantification of the resting state functional connectivity of the primary auditory (BA41) and visual areas (presented in Figure 6), at baseline and 3 years post–retinal gene therapy.

Resting state fMRI connectivity results (Figure 6)	FFX* (LH) mm^2^	FFX (RH) mm^2^	RFX^†^ (LH) mm^2^	RFX (RH) mm^2^
Baseline	1,507.65	1,672.08	97.97	162.06
3 years	2,570.5	2,934.97	361	462.82

*FFX = fixed effect. ^†^RFX = random effect. LH, left hemisphere; RH, right hemisphere.

### Connectivity of the Primary Visual Cortex to the BA41 Areas

**FIGURE 7:**
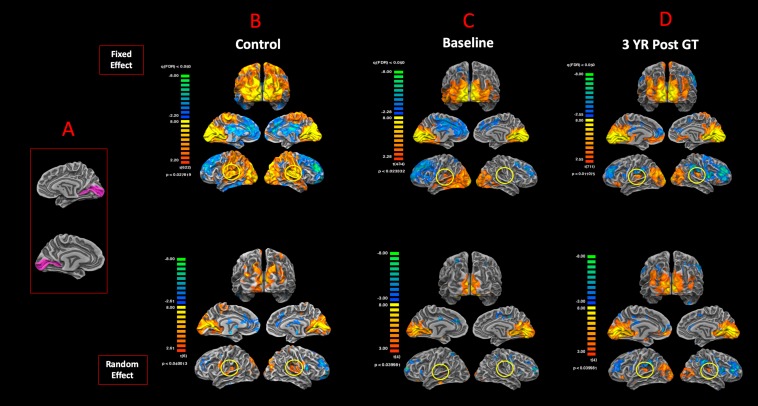
Functional connectivity of the primary visual areas. **(A)** Left and right Brodmann primary visual areas (BA17) as anatomical seed points for functional connectivity analyses. Results for the FX and RFX group analyses are shown for **(B)** sighted controls, **(C)** RPE65 patients at baseline, and **(D)** RPE65 patients 3 years post–gene therapy. All FX group analyses were performed at FDR-corrected *p* < 0.02 (*q* < 0.05), with an extent threshold of >100 mm^2^, and all RFX analyses were performed at an uncorrected *p* < 0.04 with an extent threshold >100 mm^2^. Sighted controls presented significant extended functional connectivity between the left and right BA17 areas and other brain regions, particularly for the primary auditory areas (circled yellow). Connectivity pattern for the RPE65 patients at baseline showed significant functional connectivity across the left and right visual cortex, but considerably reduced levels of connectivity between the primary visual and primary auditory cortices (circled yellow). However, 3 years after gene therapy, functional connectivity between the primary visual to primary auditory cortices significantly increased compared to baseline, but did not reach the same magnitude as observed in sighted controls.

To examine the degree of reciprocity for the functional connectivity of the visual and auditory areas, BA17 was used as the anatomical seed areas to assess the functional connectivity of the primary visual cortex to the rest of the brain. Figure 7A shows the anatomical ROIs for BA17 for both hemispheres. The FFX and RFX rsfMRI group results for the sighted controls showed significant functional connectivity between the left and right BA17 areas and other brain regions, in particular BA41 (circled in yellow) and other cortical areas dedicated to the auditory functions (Figure 7B). As compared to sighted controls, the RPE65 patients at baseline did not show extensive connectivity between BA17 and the rest of their brains, which was particularly evident in the FFX effect group results (Figure 7C). However, at the same statistical thresholds, 3 years following the subretinal injection of their second eye, RPE65 patients presented with increased connectivity within the primary visual areas, as well as increased connectivity between BA17 and BA41 (circled in yellow) (Figure 7D). Overall, the rsfMRI results showed a significant increase in the connectivity of the visual cortex to BA41 3 years after FO GT compared to baseline.

## Discussion

Performing task-based fMRI and rsfMRI on a group of visually impaired patients who regained their sight by means of retinal GT, we found that vision restoration resulted in enhancement rather than elimination of the cross-modal auditory processing. Furthermore, occupation of the visual cortex by the auditory functions did not prevent the newly recovered visual functions from occupying the same brain regions. The findings are novel because of our unique access to a group of RPE65 patients with newly recovered vision (Maguire et al., 2008; Bennett et al., 2016; Ashtari et al., 2017), not previously available to other research centers. The findings are important for understanding the mechanisms of brain plasticity and for decision to undergo retinal intervention to reverse blindness, despite the persistence of cross-modal plasticity, which has been previously cautioned to affect the success of sight restoration (Dormal et al., 2015; Legge and Chung, 2016).

The first question to address was whether the auditory cross-modal component was present in the low-vision RPE65 patients, taking into account that these patients are not completely blind at birth. Comparison of the auditory fMRI results from sighted controls (Figure 3A) to those of the RPE65 patients (Figures 3B,C) clearly demonstrates that, in line with previous reports on the early- and late-blind subjects, RPE65 patients did engage a compensatory mechanism for processing auditory stimuli. Further examination of the results from individual subjects (Supplementary Figures S1, S2) showed that auditory stimulations significantly activated the visual cortex in all but one patient (CH12) and none but one control individual (SC02 with slight visual cortex activation).

Interestingly, CH12, who was the oldest participant (44 years old), had severely impaired (near blind) vision for the ∼15 years preceding her enrollment in the study due to the retinal degenerative component of the disease. With visual function resembling that of the blind patients, CH12 was expected to show a greater participation of the visual cortex in auditory processing, as a compensatory mechanism for the vision loss. The absence of the cross-modal auditory processing in CH12 indicates that the takeover of the visual cortex by other senses may indeed require the presence of viable visual neurons, which may have been absent in this patient. Therefore, in RPE65 patients who were born with some residual visual capabilities, unlike in individuals who were born completely blind, the auditory cross-modal plasticity alone is not strong enough to support viability of visual neurons.

The second question to address was whether the auditory cross-modal component survives the vision restoration in RPE65 patients. Three years after receiving retinal GT to the contralateral eyes, the same group of RPE65 patients underwent fMRI performing the same visual and auditory tasks. The purpose was to evaluate the lasting effects of GT on the patients’ visual functions, as well as the fate of cross-modal plasticity upon the return of vision to the visual cortex. Surprisingly, the cross-modal component not only persisted 3 years after vision restoration in RPE65 patients, but also the cortical activations of the visual cortex increased in intensity (Figure 3C). This increase was highly significant within the auditory (unimodal) and visual cortex (cross-modal), when comparing auditory task performances of the RPE65 patients across time (Figure 4A).

Next, an important question to address was whether the persistence of the auditory function in visual cortex would have an adaptive or maladaptive effect on the success of vision restoration. In other words, would the auditory cross-modal nature be detrimental to the success of retinal GT 3 years after initial administration? As previously reported (Ashtari et al., 2017), and also based on comparison of fMRI responses to visual stimulation before and 3 years after GT (Figures 5B,C) and the group statistical differences between the two time points (Figures 4B,C), the visual function in RPE65 patients was robust and durable long after the retinal intervention. The apparent increase in the level of visual activity 3 years after GT, particularly for patients’ fMRI response from the newly treated eye (left eye for 7/8 patients; FO clinical trial), is thought to be due to an increase in the visual signals from the surviving photoreceptors that were revived by retinal GT (Bennett et al., 2016; Ashtari et al., 2017). The newly established visual signals from the retina actively stimulated the visual cortex, which in turn restored the functionality of the remaining viable visual neurons or assisted in unmasking dormant visual neurons to meet the greater demand on visual signal processing. This hypothesis is based on the widely demonstrated notions of brain malleability in reorganizing its circuitry in response to changes of a sensory input (Sabel et al., 2018).

To further substantiate the results from our task-based fMRI experiments, sighted controls at baseline and RPE65 patients at two time points (before and 3 years after GT) underwent rsfMRI to assess the effects of visual restoration on brain’s resting state functional connectivity (RSFC) between the auditory and visual cortices. The group results for sighted controls, using the BA41 and BA17 as seed areas, showed significant connectivity between the temporal and occipital cortex (Figure 6B) and vice versa (Figure 7B). This was unexpected, as our earlier results from the task-based auditory (Figure 3A) and visual (Figure 5A) fMRI paradigms did not show any positively correlated occipitotemporal activation for sighted controls. Here, although the RSFC results showed bidirectional functional connectivity between the auditory and visual cortices, no cross activations were observed when the sighted control subjects performed the task-based fMRI experiments (Figures 3A, 5A). The results from the RSFC and the auditory task-based fMRI presented here are consistent with those reported in a recent study by Pelland et al. (2017). According to a report by Iurilli et al. (2012) in sighted controls with no visual impairment, the effect of auditory stimulation on the visual cortex is mediated by a local GABAergic inhibitory circuit (Iurilli et al., 2012). Thus, despite the observed significant RSFC between the auditory and visual areas among sighted controls (Figure 6B), as depicted in Figure 3A, auditory stimulation did not result in cortical activations of the visual cortex (Iurilli et al., 2012). In support of RSFC results, direct projections from the primary auditory to the primary visual cortex areas have also been reported in normal primates (Falchier et al., 2002; Rockland and Ojima, 2003), normal-sighted cats (Fishman and Michael, 1973; Innocenti et al., 1988), and retrograde tracer injection studies in congenitally deaf cats demonstrating increased anatomic connectivity between the auditory and visual cortex, as substrate for cross-modal reorganization, which was not observed in normal-hearing cats (Barone et al., 2013; Butler et al., 2017) and using the diffusion tensor imaging in normal-sighted humans (Eckert et al., 2008; Beer et al., 2011; Chen et al., 2013). Additional support for the presence of a direct connectivity between the auditory and visual areas comes from modeling how a non-visual information reaches the occipital cortex in blind and sighted controls using an auditory discrimination paradigm (Klinge et al., 2010). Authors reported that the model that assumes bidirectional connections between the medial geniculate nucleus (MGN), primary auditory cortex (BA41), and primary visual cortex (BA17) outperformed all other models in both groups (Klinge et al., 2010). In addition, the corticocortical connections from the BA41 to the BA17 were reported to be much stronger as compared to the thalamocortical connections, such as MGN to V1. As a final conclusion, we selected direct cortical connections between the BA41 and BA17 to be the most probable connection that facilitates the auditory information evoking responses in the primary visual cortex of blind individuals. Together, the existing reports on sighted controls are suggestive of an intrinsic connectivity between the occipital and temporal cortex, which is shown to be unmasked upon demands from other sensory inputs. Examples for such unmasking are the studies of artificially induced short-term visual deprivation in sighted controls that have resulted in on-demand but transient enhancement of other senses (Lewald, 2007; Merabet et al., 2008).

Similar to the sighted controls, we observed significant RSFC between the temporal and occipital cortex in the RPE65 patients, albeit more attenuated than controls, particularly at baseline than 3 years after retinal GT. Contrary to the sighted controls, the auditory stimulation of the RPE65 patients showed highly significant cross-modal cortical activations of the visual cortex (Figures 3B), which were considerably enhanced after retinal GT (Figure 3C). These results also are suggestive of the fact that the local GABAergic inhibitory circuit (Iurilli et al., 2012) may be dysfunctional in these patients. We hypothesize that in the absence of visually evoked responses of visual neurons the sound-driven inhibition might have faded away because it is of no use regardless of the direction or orientation tuning. On the contrary, the excitation-mediated recruitment of visual neurons potentiates the capabilities of auditory processing and therefore might be reinforced by cross-modal plasticity. These results further support the notion of on-demand unmasking and perhaps strengthening of a preexisting connectivity between the auditory and visual cortex for the low-vision RPE65 patients. We also hypothesize that the posttherapy potentiation of auditory fMRI activations in the visual cortex (Figure 4) resulted from the revitalizing of the neurovascular coupling in response to the increased energy demand by visual neurons (Sabel et al., 2018). The enhanced RSFC after retinal GT is thought to be due to increased availability of the visual neurons and increased blood flow to these neurons set forth by the newly established visual signals from the retina along with continuous stimulation of the visual system over time.

Although the underlying process of the cross-modal plasticity is largely unknown, a recent review by Kupers and Ptito (2014) proposed two separate hypotheses for the formation of the cross-modal plasticity. The first hypothesis assumes development of new pathways within the deprived brain (visual cortex), and the second one assumes the unmasking and strengthening of existing connections. Based on the aforementioned evidence and our preliminary results, we favor the second hypothesis suggested by Kupers and Ptito as the basis for the auditory cross-modal formation in the RPE65 patients. In addition, our results show that upon the return of vision to the visual cortex this connectivity has not only persisted but rather enhanced.

## Conclusion

Taken together, our results are suggestive of the fact that visually impaired RPE65 patients, similar to blind individuals, develop cross-modal plasticity for auditory functions and their visual cortex successfully responds to both the visual and auditory stimuli. Upon partial vision restoration, not only the auditory cross-modal component persisted in the visual cortex of RPE65 patients, but also the connectivity between auditory and visual cortices was significantly enhanced, supported by both the RSFC and task-based fMRI experiments. More importantly, our data supports the fact that the overall success of retinal GT is not hindered by the presence of auditory cross-modal plasticity, and the visual cortex remains sensitive to both visual and auditory stimulations, even 3 years after the return of vision.

Although some aspects of vision may be affected by the occupation of the visual cortex by auditory cross-modal plasticity, our experimental design was not sensitive to show such effects. Additional studies evaluating low-vision patients before and after retinal intervention to restore sight along with more sophisticated auditory/visual stimulations are essential in extending the findings presented here to further validate the presence and persistence of cross-modal plasticity post retinal intervention. Importantly, elaborate studies are needed to identify the visual functions that are affected by other non-visual cross-modal plasticity in the visual cortex. Our up-to-date findings show that regardless of visual cortex lending itself to non-visual auditory sense during long-term visual deprivation, the retinal GT successfully recovers the visual functions in the visual cortex.

## Data Availability Statement

All datasets generated for this study are included in the article/Supplementary Material.

## Ethics Statement

The studies involving human participants were reviewed and approved by Children’s Hospital of Philadelphia IRB. Written informed consent to participate in this study was provided by the participants’ legal guardian/next of kin.

## Author Contributions

TM, AW, and MM: recruitment of study participants, performed fMRI experiments, processed the experimental data, performed the analysis, contributed to the manuscript, drafted the manuscript, analysis of the results, and to the writing of the manuscript. TM: compiled the references for the entire manuscript. ML: provided critical feedback on the results and conclusions and helped in writing and editing the manuscript. AH: supervised the statistical analyses of data for the project and ensuring that questions related to the accuracy or integrity of any part. AM and JB: patient referral, overall support of the project, manuscript editing, and contributed to the final version of the manuscript. MA: developed the main conceptual design and theory of the study, experimental design, directed the project, supervised the data analyses and interpretation, writing of the manuscript, held critical discussions on the results and conclusions with authors, edited manuscript, and final manuscript approval.

## Conflict of Interest

JB and AM were co-inventors of a method to treat or slow the development of blindness (US patent number 8147823), but both waived any financial interest in this technology in 2002. JB was a co-founder of Gensight Biologics and Limelight Bio and a scientific (non-equity-holding) founder of Spark Therapeutics. JB was also a co-author of several provisional patents relevant to gene therapy for retinal degeneration. AM was the PI of two Clinical Trial Agreements with Spark Therapeutics. The remaining authors declare that the research was conducted in the absence of any commercial or financial relationships that could be construed as a potential conflict of interest.

## Footnotes

1 www.clinicaltrials.gov

2 www.mrivideo.com

3 www.pstnet.com/eprime.cfm

4 www.brainvoyager.com

## References

[B1] Almeida J. He D. Chen Q. Mahon B. Z. Zhang F. Goncalves O. F. (2015). Decoding visual location from neural patterns in the auditory cortex of the congenitally deaf. *Psychol. Sci.* 26 1771–1782. 10.1177/0956797615598970 26423461 PMC5209787

[B2] Amedi A. Raz N. Azulay H. Malach R. Zohary E. (2010). Cortical activity during tactile exploration of objects in blind and sighted humans. *Restor. Neurol. Neurosci.* 28 143–156. 10.3233/RNN-2010-0503 20404404

[B3] Amedi A. Stern W. M. Camprodon J. A. Bermpohl F. Merabet L. Rotman S. (2007). Shape conveyed by visual-to-auditory sensory substitution activates the lateral occipital complex. *Nat. Neurosci.* 10 687–689. 10.1038/nn1912 17515898

[B4] Anderson C. A. Wiggins I. M. Kitterick P. T. Hartley D. E. H. (2017). Adaptive benefit of cross-modal plasticity following cochlear implantation in deaf adults. *Proc. Natl. Acad. Sci. U.S.A.* 114 10256–10261. 10.1073/pnas.1704785114 28808014 PMC5617272

[B5] Ashtari M. Cyckowski L. Yazdi A. Viands A. Marshall K. Bokkon I. (2014). fMRI of retina-originated phosphenes experienced by patients with Leber congenital amaurosis. *PLoS One* 9:e86068. 10.1371/journal.pone.0086068 24465873 PMC3897613

[B6] Ashtari M. Cyckowski L. L. Monroe J. F. Marshall K. A. Chung D. C. Auricchio A. (2011). The human visual cortex responds to gene therapy-mediated recovery of retinal function. *J. Clin. Invest.* 121 2160–2168. 10.1172/JCI57377 21606598 PMC3104779

[B7] Ashtari M. Nikonova E. S. Marshall K. A. Young G. J. Aravand P. Pan W. (2017). The Role of the Human Visual Cortex in Assessment of the Long-Term Durability of Retinal Gene Therapy in Follow-on RPE65 Clinical Trial Patients. *Ophthalmology* 124 873–883. 10.1016/j.ophtha.2017.01.029 28237426 PMC5805133

[B8] Barone P. Lacassagne L. Kral A. (2013). Reorganization of the connectivity of cortical field DZ in congenitally deaf cat. *PLoS One* 8:e60093. 10.1371/journal.pone.0060093 23593166 PMC3625188

[B9] Bavelier D. Neville H. J. (2002). Cross-modal plasticity: where and how? *Nat. Rev. Neurosci.* 3 443–452. 10.1038/nrn848 12042879

[B10] Bedny M. Konkle T. Pelphrey K. Saxe R. Pascual-Leone A. (2010). Sensitive period for a multimodal response in human visual motion area MT/MST. *Curr. Biol.* 20 1900–1906. 10.1016/j.cub.2010.09.044 20970337 PMC2998392

[B11] Bedny M. Pascual-Leone A. Dodell-Feder D. Fedorenko E. Saxe R. (2011). Language processing in the occipital cortex of congenitally blind adults. *Proc. Natl. Acad. Sci. U.S.A.* 108 4429–4434. 10.1073/pnas.1014818108 21368161 PMC3060248

[B12] Beer A. L. Plank T. Greenlee M. W. (2011). Diffusion tensor imaging shows white matter tracts between human auditory and visual cortex. *Exp. Brain Res.* 213 299–308. 10.1007/s00221-011-2715-y 21573953

[B13] Bennett J. Ashtari M. Wellman J. Marshall K. A. Cyckowski L. L. Chung D. C. (2012). AAV2 gene therapy readministration in three adults with congenital blindness. *Sci. Transl. Med.* 4:120ra115. 10.1126/scitranslmed.3002865 22323828 PMC4169122

[B14] Bennett J. Wellman J. Marshall K. A. McCague S. Ashtari M. DiStefano-Pappas J. (2016). Safety and durability of effect of contralateral-eye administration of AAV2 gene therapy in patients with childhood-onset blindness caused by RPE65 mutations: a follow-on phase 1 trial. *Lancet* 388 661–672. 10.1016/S0140-6736(16)30371-3 27375040 PMC5351775

[B15] Biswal B. Yetkin F. Z. Haughton V. M. Hyde J. S. (1995). Functional connectivity in the motor cortex of resting human brain using echo-planar MRI. *Magn. Reson. Med.* 34 537–541. 10.1002/mrm.1910340409 8524021

[B16] Bola M. Borchardt V. (2016). Cognitive Processing Involves Dynamic Reorganization of the Whole-Brain Network’s Functional Community Structure. *J. Neurosci.* 36 3633–3635. 10.1523/JNEUROSCI.0106-16.2016 27030750 PMC6601742

[B17] Bonino D. Ricciardi E. Sani L. Gentili C. Vanello N. Guazzelli M. (2008). Tactile spatial working memory activates the dorsal extrastriate cortical pathway in congenitally blind individuals. *Arch. Ital. Biol.* 146 133–146. 19378878

[B18] Bowne S. J. Humphries M. M. Sullivan L. S. Kenna P. F. Tam L. C. Kiang A. S. (2011). A dominant mutation in RPE65 identified by whole-exome sequencing causes retinitis pigmentosa with choroidal involvement. *Eur. J. Hum. Genet.* 19 1074–1081. 10.1038/ejhg.2011.86 21654732 PMC3190249

[B19] Buckley K. A. Tobey E. A. (2011). Cross-modal plasticity and speech perception in pre- and postlingually deaf cochlear implant users. *Ear Hear* 32 2–15. 10.1097/AUD.0b013e3181e8534c 20829699

[B20] Burton H. (2003). Visual cortex activity in early and late blind people. *J. Neurosci.* 23 4005–4011. 10.1523/jneurosci.23-10-04005.2003 12764085 PMC3667661

[B21] Burton H. Snyder A. Z. Diamond J. B. Raichle M. E. (2002). Adaptive changes in early and late blind: a FMRI study of verb generation to heard nouns. *J. Neurophysiol.* 88 3359–3371. 10.1152/jn.00129.2002 12466452 PMC3704164

[B22] Butler B. E. Chabot N. Kral A. Lomber S. G. (2017). Origins of thalamic and cortical projections to the posterior auditory field in congenitally deaf cats. *Hear. Res.* 343 118–127. 10.1016/j.heares.2016.06.003 27306930

[B23] Calhoun V. D. Adali T. Pekar J. J. Pearlson G. D. (2003). Latency (in)sensitive ICA. Group independent component analysis of fMRI data in the temporal frequency domain. *Neuroimage* 20 1661–1669. 10.1016/s1053-8119(03)00411-7 14642476

[B24] Campbell J. Sharma A. (2014). Cross-modal re-organization in adults with early stage hearing loss. *PLoS One* 9:e90594. 10.1371/journal.pone.0090594 24587400 PMC3938766

[B25] Cattaneo Z. Lega C. Cecchetto C. Papagno C. (2014). Auditory deprivation affects biases of visuospatial attention as measured by line bisection. *Exp. Brain Res.* 232 2767–2773. 10.1007/s00221-014-3960-7 24770861

[B26] Cecere R. Romei V. Bertini C. Ladavas E. (2014). Crossmodal enhancement of visual orientation discrimination by looming sounds requires functional activation of primary visual areas: a case study. *Neuropsychologia* 56 350–358. 10.1016/j.neuropsychologia.2014.02.008 24534140

[B27] Chen L. C. Stropahl M. Schonwiesner M. Debener S. (2017). Enhanced visual adaptation in cochlear implant users revealed by concurrent EEG-fNIRS. *Neuroimage* 146 600–608. 10.1016/j.neuroimage.2016.09.033 27640748

[B28] Chen Z. Li J. Liu M. Ma L. (2013). [Structural connectivity between visual cortex and auditory cortex in healthy adults: a diffusion tensor imaging study]. *Nan Fang Yi Ke Da Xue Xue Bao* 33 338–341. 23529227

[B29] Collignon O. Champoux F. Voss P. Lepore F. (2011a). Sensory rehabilitation in the plastic brain. *Prog. Brain Res.* 191 211–231. 10.1016/B978-0-444-53752-2.00003-5 21741554

[B30] Collignon O. Vandewalle G. Voss P. Albouy G. Charbonneau G. Lassonde M. (2011b). Functional specialization for auditory-spatial processing in the occipital cortex of congenitally blind humans. *Proc. Natl. Acad. Sci. U.S.A.* 108 4435–4440. 10.1073/pnas.1013928108 21368198 PMC3060256

[B31] Collignon O. Dormal G. Albouy G. Vandewalle G. Voss P. Phillips C. (2013). Impact of blindness onset on the functional organization and the connectivity of the occipital cortex. *Brain* 136(Pt 9), 2769–2783. 10.1093/brain/awt176 23831614

[B32] Collignon O. Lassonde M. Lepore F. Bastien D. Veraart C. (2007). Functional cerebral reorganization for auditory spatial processing and auditory substitution of vision in early blind subjects. *Cereb. Cortex* 17 457–465. 10.1093/cercor/bhj162 16581983

[B33] Collignon O. Voss P. Lassonde M. Lepore F. (2009). Cross-modal plasticity for the spatial processing of sounds in visually deprived subjects. *Exp. Brain Res.* 192 343–358. 10.1007/s00221-008-1553-z 18762928

[B34] den Hollander A. I. Roepman R. Koenekoop R. K. Cremers F. P. (2008). Leber congenital amaurosis: genes, proteins and disease mechanisms. *Prog. Retin. Eye Res.* 27 391–419. 10.1016/j.preteyeres.2008.05.003 18632300

[B35] Dewey R. S. Hartley D. E. (2015). Cortical cross-modal plasticity following deafness measured using functional near-infrared spectroscopy. *Hear. Res.* 325 55–63. 10.1016/j.heares.2015.03.007 25819496

[B36] Dormal G. Lepore F. Harissi-Dagher M. Albouy G. Bertone A. Rossion B. (2015). Tracking the evolution of crossmodal plasticity and visual functions before and after sight restoration. *J. Neurophysiol.* 113 1727–1742. 10.1152/jn.00420.2014 25520432 PMC4359990

[B37] Dormal G. Rezk M. Yakobov E. Lepore F. Collignon O. (2016). Auditory motion in the sighted and blind: early visual deprivation triggers a large-scale imbalance between auditory and “visual” brain regions. *Neuroimage* 134 630–644. 10.1016/j.neuroimage.2016.04.027 27107468

[B38] Doucet M. E. Bergeron F. Lassonde M. Ferron P. Lepore F. (2006). Cross-modal reorganization and speech perception in cochlear implant users. *Brain* 129(Pt 12), 3376–3383. 10.1093/brain/awl264 17003067

[B39] Eckert M. A. Kamdar N. V. Chang C. E. Beckmann C. F. Greicius M. D. Menon V. (2008). A cross-modal system linking primary auditory and visual cortices: evidence from intrinsic fMRI connectivity analysis. *Hum. Brain Mapp.* 29 848–857. 10.1002/hbm.20560 18412133 PMC2605422

[B40] Falchier A. Clavagnier S. Barone P. Kennedy H. (2002). Anatomical evidence of multimodal integration in primate striate cortex. *J. Neurosci.* 22 5749–5759. 10.1523/jneurosci.22-13-05749.2002 12097528 PMC6758216

[B41] Fischl B. Sereno M. I. Dale A. M. (1999). Cortical surface-based analysis. II: inflation, flattening, and a surface-based coordinate system. *Neuroimage* 9 195–207. 10.1006/nimg.1998.0396 9931269

[B42] Fishman M. C. Michael P. (1973). Integration of auditory information in the cat’s visual cortex. *Vis. Res.* 13 1415–1419. 10.1016/0042-6989(73)90002-3 4719075

[B43] Frasnelli J. Collignon O. Voss P. Lepore F. (2011). Crossmodal plasticity in sensory loss. *Prog. Brain Res.* 191 233–249. 10.1016/B978-0-444-53752-2.00002-3 21741555

[B44] Friston K. J. Fletcher P. Josephs O. Holmes A. Rugg M. D. Turner R. (1998). Event-related fMRI: characterizing differential responses. *Neuroimage* 7 30–40. 10.1006/nimg.1997.0306 9500830

[B45] Genovese C. R. Lazar N. A. Nichols T. (2002). Thresholding of statistical maps in functional neuroimaging using the false discovery rate. *Neuroimage* 15 870–878. 10.1006/nimg.2001.1037 11906227

[B46] Giraud A. L. Price C. J. Graham J. M. Frackowiak R. S. (2001). Functional plasticity of language-related brain areas after cochlear implantation. *Brain* 124(Pt 7), 1307–1316. 10.1093/brain/124.7.1307 11408326

[B47] Glick H. Sharma A. (2017). Cross-modal plasticity in developmental and age-related hearing loss: clinical implications. *Hear. Res.* 343 191–201. 10.1016/j.heares.2016.08.012 27613397 PMC6590524

[B48] Goebel R. Esposito F. Formisano E. (2006). Analysis of functional image analysis contest (FIAC) data with brainvoyager QX: from single-subject to cortically aligned group general linear model analysis and self-organizing group independent component analysis. *Hum. Brain Mapp.* 27 392–401. 10.1002/hbm.20249 16596654 PMC6871277

[B49] Gougoux F. Belin P. Voss P. Lepore F. Lassonde M. Zatorre R. J. (2009). Voice perception in blind persons: a functional magnetic resonance imaging study. *Neuropsychologia* 47 2967–2974. 10.1016/j.neuropsychologia.2009.06.027 19576235

[B50] Gougoux F. Zatorre R. J. Lassonde M. Voss P. Lepore F. (2005). A functional neuroimaging study of sound localization: visual cortex activity predicts performance in early-blind individuals. *PLoS Biol.* 3:e27. 10.1371/journal.pbio.0030027 15678166 PMC544927

[B51] Goyal M. S. Hansen P. J. Blakemore C. B. (2006). Tactile perception recruits functionally related visual areas in the late-blind. *Neuroreport* 17 1381–1384. 10.1097/01.wnr.0000227990.23046.fe 16932143

[B52] Green M. F. Glahn D. Engel S. A. Nuechterlein K. H. Sabb F. Strojwas M. (2005). Regional brain activity associated with visual backward masking. *J. Cogn. Neurosci.* 17 13–23. 10.1162/0898929052880011 15701236

[B53] Guerreiro M. J. S. Putzar L. Roder B. (2016). Persisting cross-modal changes in sight-recovery individuals modulate visual perception. *Curr. Biol.* 26 3096–3100. 10.1016/j.cub.2016.08.069 27746025

[B54] Heimler B. Weisz N. Collignon O. (2014). Revisiting the adaptive and maladaptive effects of crossmodal plasticity. *Neuroscience* 283 44–63. 10.1016/j.neuroscience.2014.08.003 25139761

[B55] Innocenti G. M. Berbel P. Clarke S. (1988). Development of projections from auditory to visual areas in the cat. *J. Comp. Neurol.* 272 242–259. 10.1002/cne.902720207 2456313

[B56] Iraji A. Chen H. Wiseman N. Zhang T. Welch R. O’Neil B. (2016). Connectome-scale assessment of structural and functional connectivity in mild traumatic brain injury at the acute stage. *Neuroimage Clin.* 12 100–115. 10.1016/j.nicl.2016.06.012 27408795 PMC4932612

[B57] Iurilli G. Ghezzi D. Olcese U. Lassi G. Nazzaro C. Tonini R. (2012). Sound-driven synaptic inhibition in primary visual cortex. *Neuron* 73 814–828. 10.1016/j.neuron.2011.12.026 22365553 PMC3315003

[B58] Jiang F. Stecker G. C. Boynton G. M. Fine I. (2016). Early blindness results in developmental plasticity for auditory motion processing within auditory and occipital cortex. *Front. Hum. Neurosci.* 10:324. 10.3389/fnhum.2016.00324 27458357 PMC4932114

[B59] Jin M. Li S. Moghrabi W. N. Sun H. Travis G. H. (2005). Rpe65 is the retinoid isomerase in bovine retinal pigment epithelium. *Cell* 122 449–459. 10.1016/j.cell.2005.06.042 16096063 PMC2748856

[B60] Klinge C. Eippert F. Roder B. Buchel C. (2010). Corticocortical connections mediate primary visual cortex responses to auditory stimulation in the blind. *J. Neurosci.* 30 12798–12805. 10.1523/JNEUROSCI.2384-10.2010 20861384 PMC6633575

[B61] Kral A. Dorman A. F. Wilson B. S. (2019). Neuronal development of hearing and language: cochlear implants and critical periods. *Annu. Rev. Neurosci.* 42 47–65. 10.1146/annurev-neuro-080317-61513 30699049

[B62] Kral A. Sharma A. (2012). Developmental neuroplasticity after cochlear implantation. *Trends Neurosci.* 35 111–122. 10.1016/j.tins.2011.09.004 22104561 PMC3561718

[B63] Kriegeskorte N. Goebel R. (2001). An efficient algorithm for topologically correct segmentation of the cortical sheet in anatomical mr volumes. *Neuroimage* 14 329–346. 10.1006/nimg.2001.0831 11467907

[B64] Kumaran N. Moore A. T. Weleber R. G. Michaelides M. (2017). Leber congenital amaurosis/early-onset severe retinal dystrophy: clinical features, molecular genetics and therapeutic interventions. *Br. J. Ophthalmol.* 101 1147–1154. 10.1136/bjophthalmol-2016-309975 28689169 PMC5574398

[B65] Kupers R. Ptito M. (2014). Compensatory plasticity and cross-modal reorganization following early visual deprivation. *Neurosci. Biobehav. Rev.* 41 36–52. 10.1016/j.neubiorev.2013.08.001 23954750

[B66] Land R. Baumhoff P. Tillein J. Lomber S. G. Hubka P. Kral A. (2016). Cross-modal plasticity in higher-order auditory cortex of congenitally deaf cats does not limit auditory responsiveness to cochlear implants. *J. Neurosci.* 36 6175–6185. 10.1523/JNEUROSCI.0046-16.2016 27277796 PMC4899523

[B67] Legge G. E. Chung S. T. L. (2016). Low vision and plasticity: implications for rehabilitation. *Annu. Rev. Vis. Sci.* 2 321–343. 10.1146/annurev-vision-111815-114344 28532346 PMC7792632

[B68] Leo A. Bernardi G. Handjaras G. Bonino D. Ricciardi E. Pietrini P. (2012). Increased BOLD variability in the parietal cortex and enhanced parieto-occipital connectivity during tactile perception in congenitally blind individuals. *Neural Plast.* 2012:720278. 10.1155/2012/720278 22792493 PMC3388315

[B69] Lewald J. (2007). More accurate sound localization induced by short-term light deprivation. *Neuropsychologia* 45 1215–1222. 10.1016/j.neuropsychologia.2006.10.006 17113113

[B70] Lowe M. J. Mock B. J. Sorenson J. A. (1998). Functional connectivity in single and multislice echoplanar imaging using resting state fluctuations. *Neuroimage* 7 119–132. 10.1006/nimg.1997.0315 9558644

[B71] Lyness C. R. Woll B. Campbell R. Cardin V. (2013). How does visual language affect crossmodal plasticity and cochlear implant success? *Neurosci. Biobehav. Rev.* 37(10 Pt 2), 2621–2630. 10.1016/j.neubiorev.2013.08.011 23999083 PMC3989033

[B72] Maguire A. M. Simonelli F. Pierce E. A. Pugh E. N. Jr. Mingozzi F. (2008). Safety and efficacy of gene transfer for Leber’s congenital amaurosis. *N. Engl. J. Med.* 358 2240–2248. 10.1056/NEJMoa0802315 18441370 PMC2829748

[B73] Merabet L. Thut G. Murray B. Andrews J. Hsiao S. Pascual-Leone A. (2004). Feeling by sight or seeing by touch? *Neuron* 42 173–179. 10.1016/s0896-6273(04)00147-3 15066274

[B74] Merabet L. B. Hamilton R. Schlaug G. Swisher J. D. Kiriakopoulos E. T. Pitskel N. B. (2008). Rapid and reversible recruitment of early visual cortex for touch. *PLoS One* 3:e3046. 10.1371/journal.pone.0003046 18728773 PMC2516172

[B75] Moiseyev G. Chen Y. Takahashi Y. Wu B. X. Ma J. X. (2005). RPE65 is the isomerohydrolase in the retinoid visual cycle. *Proc. Natl. Acad. Sci. U.S.A.* 102 12413–12418. 10.1073/pnas.0503460102 16116091 PMC1194921

[B76] Noppeney U. Friston K. J. Price C. J. (2003). Effects of visual deprivation on the organization of the semantic system. *Brain* 126(Pt 7), 1620–1627. 10.1093/brain/awg152 12805112

[B77] Oh S. H. Kim C. S. Kang E. J. Lee D. S. Lee H. J. Chang S. O. (2003). Speech perception after cochlear implantation over a 4-year time period. *Acta Otolaryngol.* 123 148–153. 10.1080/0036554021000028111 12701730

[B78] Pasqualotto A. Proulx M. J. (2013). The study of blindness and technology can reveal the mechanisms of three-dimensional navigation. *Behav. Brain Sci.* 36 559–560. 10.1017/S0140525X13000496 24103614

[B79] Pelland M. Orban P. Dansereau C. Lepore F. Bellec P. Collignon O. (2017). State-dependent modulation of functional connectivity in early blind individuals. *Neuroimage* 147 532–541. 10.1016/j.neuroimage.2016.12.053 28011254

[B80] Pietrini P. Furey M. L. Ricciardi E. Gobbini M. I. Wu W. H. Cohen L. (2004). Beyond sensory images: object-based representation in the human ventral pathway. *Proc. Natl. Acad. Sci. U.S.A.* 101 5658–5663. 10.1073/pnas.0400707101 15064396 PMC397466

[B81] Ptito M. Schneider F. C. Paulson O. B. Kupers R. (2008). Alterations of the visual pathways in congenital blindness. *Exp. Brain Res.* 187 41–49. 10.1007/s00221-008-1273-4 18224306

[B82] Raz N. Amedi A. Zohary E. (2005). V1 activation in congenitally blind humans is associated with episodic retrieval. *Cereb. Cortex* 15 1459–1468. 10.1093/cercor/bhi026 15647525

[B83] Redmond T. M. Poliakov E. Yu S. Tsai J. Y. Lu Z. Gentleman S. (2005). Mutation of key residues of RPE65 abolishes its enzymatic role as isomerohydrolase in the visual cycle. *Proc. Natl. Acad. Sci. U.S.A.* 102 13658–13663. 10.1073/pnas.0504167102 16150724 PMC1224626

[B84] Redmond T. M. Yu S. Lee E. Bok D. Hamasaki D. Chen N. (1998). Rpe65 is necessary for production of 11-cis-vitamin A in the retinal visual cycle. *Nat. Genet.* 20 344–351. 10.1038/3813 9843205

[B85] Reich L. Szwed M. Cohen L. Amedi A. (2011). A ventral visual stream reading center independent of visual experience. *Curr. Biol.* 21 363–368. 10.1016/j.cub.2011.01.040 21333539

[B86] Renier L. De Volder A. G. Rauschecker J. P. (2014). Cortical plasticity and preserved function in early blindness. *Neurosci. Biobehav. Rev.* 41 53–63. 10.1016/j.neubiorev.2013.01.025 23453908 PMC3818399

[B87] Rockland K. S. Ojima H. (2003). Multisensory convergence in calcarine visual areas in macaque monkey. *Int. J. Psychophysiol.* 50 19–26. 10.1016/s0167-8760(03)00121-1 14511833

[B88] Roder B. Ley P. Shenoy B. H. Kekunnaya R. Bottari D. (2013). Sensitive periods for the functional specialization of the neural system for human face processing. *PNAS* 110 16760–16765. 10.1073/pnas.1309963110 24019474 PMC3801039

[B89] Roder B. Stock O. Bien S. Neville H. Rosler F. (2002). Speech processing activates visual cortex in congenitally blind humans. *Eur. J. Neurosci.* 16 930–936. 10.1046/j.1460-9568.2002.02147.x 12372029

[B90] Roder B. Teder-Salejarvi W. Sterr A. Rosler F. Hillyard S. A. Neville H. J. (1999). Improved auditory spatial tuning in blind humans. *Nature* 400 162–166. 10.1038/22106 10408442

[B91] Rouger J. Lagleyre S. Demonet J. F. Fraysse B. Deguine O. Barone P. (2012). Evolution of crossmodal reorganization of the voice area in cochlear-implanted deaf patients. *Hum. Brain Mapp.* 33 1929–1940. 10.1002/hbm.21331 21557388 PMC6870380

[B92] Sabel B. A. Flammer J. Merabet L. B. (2018). Residual vision activation and the brain-eye-vascular triad: dysregulation, plasticity and restoration in low vision and blindness - a review. *Restor. Neurol. Neurosci.* 36 767–791. 10.3233/RNN-180880 30412515 PMC6294586

[B93] Sadato N. Okada T. Honda M. Yonekura Y. (2002). Critical period for cross-modal plasticity in blind humans: a functional MRI study. *Neuroimage* 16 389–400. 10.1006/nimg.2002.1111 12030824

[B94] Sadato N. Okada T. Kubota K. Yonekura Y. (2004). Tactile discrimination activates the visual cortex of the recently blind naive to Braille: a functional magnetic resonance imaging study in humans. *Neurosci. Lett.* 359 49–52. 10.1016/j.neulet.2004.02.005 15050709

[B95] Sadato N. Pascual-Leone A. Grafman J. Ibanez V. Deiber M. P. Dold G. (1996). Activation of the primary visual cortex by Braille reading in blind subjects. *Nature* 380 526–528. 10.1038/380526a0 8606771

[B96] Saenz M. Lewis L. B. Huth A. G. Fine I. Koch C. (2008). Visual motion area MT+/V5 responds to auditory motion in human sight-recovery subjects. *J. Neurosci.* 28 5141–5148. 10.1523/JNEUROSCI.0803-08.2008 18480270 PMC3165167

[B97] Sandmann P. Dillier N. Eichele T. Meyer M. Kegel A. Pascual-Marqui R. D. (2012). Visual activation of auditory cortex reflects maladaptive plasticity in cochlear implant users. *Brain* 135(Pt 2), 555–568. 10.1093/brain/awr329 22232592

[B98] Sharma A. Campbell J. Cardon G. (2015). Developmental and cross-modal plasticity in deafness: evidence from the P1 and N1 event related potentials in cochlear implanted children. *Int. J. Psychophysiol.* 95 135–144. 10.1016/j.ijpsycho.2014.04.007 24780192 PMC4209331

[B99] Sharma A. Gilley P. M. Dorman M. F. Baldwin R. (2007). Deprivation-induced cortical reorganization in children with cochlear implants. *Int. J. Audiol.* 46 494–499. 10.1080/14992020701524836 17828665

[B100] Strelnikov K. Rouger J. Demonet J. F. Lagleyre S. Fraysse B. Deguine O. (2013). Visual activity predicts auditory recover from deafness after adult cochlear implantation. *Brain* 136 3682–3695. 10.1093/brain/awt274 24136826

[B101] Stropahl M. Plotz K. Schonfeld R. Lenarz T. Sandmann P. Yovel G. (2015). Cross-modal reorganization in cochlear implant users: auditory cortex contributes to visual face processing. *Neuroimage* 121 159–170. 10.1016/j.neuroimage.2015.07.062 26220741

[B102] Viola F. C. Thorne J. D. Bleeck S. Eyles J. Debener S. (2011). Uncovering auditory evoked potentials from cochlear implant users with independent component analysis. *Psychophysiology* 48 1470–1480. 10.1111/j.1469-8986.2011.01224.x 21635266

[B103] Voss P. (2013). Sensitive and critical periods in visual sensory deprivation. *Front. Psychol.* 4:664. 10.3389/fpsyg.2013.00664 24133469 PMC3783842

[B104] Voss P. Lassonde M. Gougoux F. Fortin M. Guillemot J. P. Lepore F. (2004). Early- and late-onset blind individuals show supra-normal auditory abilities in far-space. *Curr. Biol.* 14 1734–1738. 10.1016/j.cub.2004.09.051 15458644

[B105] Watkins K. E. Shakespeare T. J. O’Donoghue M. C. Alexander I. Ragge N. Cowey A. (2013). Early auditory processing in area V5/MT+ of the congenitally blind brain. *J. Neurosci.* 33 18242–18246. 10.1523/JNEUROSCI.2546-13.2013 24227733 PMC6619753

[B106] Weeks R. Horwitz B. Aziz-Sultan A. Tian B. Wessinger C. M. Cohen L. G. (2000). A positron emission tomographic study of auditory localization in the congenitally blind. *J. Neurosci.* 20 2664–2672. 10.1523/jneurosci.20-07-02664.2000 10729347 PMC6772250

[B107] Weissenbacher A. Kasess C. Gerstl F. Lanzenberger R. Moser E. Windischberger C. (2009). Correlations and anticorrelations in resting-state functional connectivity MRI: a quantitative comparison of preprocessing strategies. *Neuroimage* 47 1408–1416. 10.1016/j.neuroimage.2009.05.005 19442749

[B108] Wilson B. S. Dorman M. F. (2008). Cochlear implants: a remarkable past and a brilliant future. *Hear. Res.* 242 3–21. 10.1016/j.heares.2008.06.005 18616994 PMC3707130

[B109] Zangaladze A. Epstein C. M. Grafton S. T. Sathian K. (1999). Involvement of visual cortex in tactile discrimination of orientation. *Nature* 401 587–590. 10.1038/44139 10524625

