# Supplementary material for: Compensatory Cross-Modal Plasticity Persists After Sight Restoration
Source: Front Neurosci. 2020 May 12;14:291. doi: 10.3389/fnins.2020.00291 (PMC7235304; doi:10.3389/fnins.2020.00291)
Supplement: Supplementary file 1 [file Data_Sheet_1.PDF]

Supplementary Figures

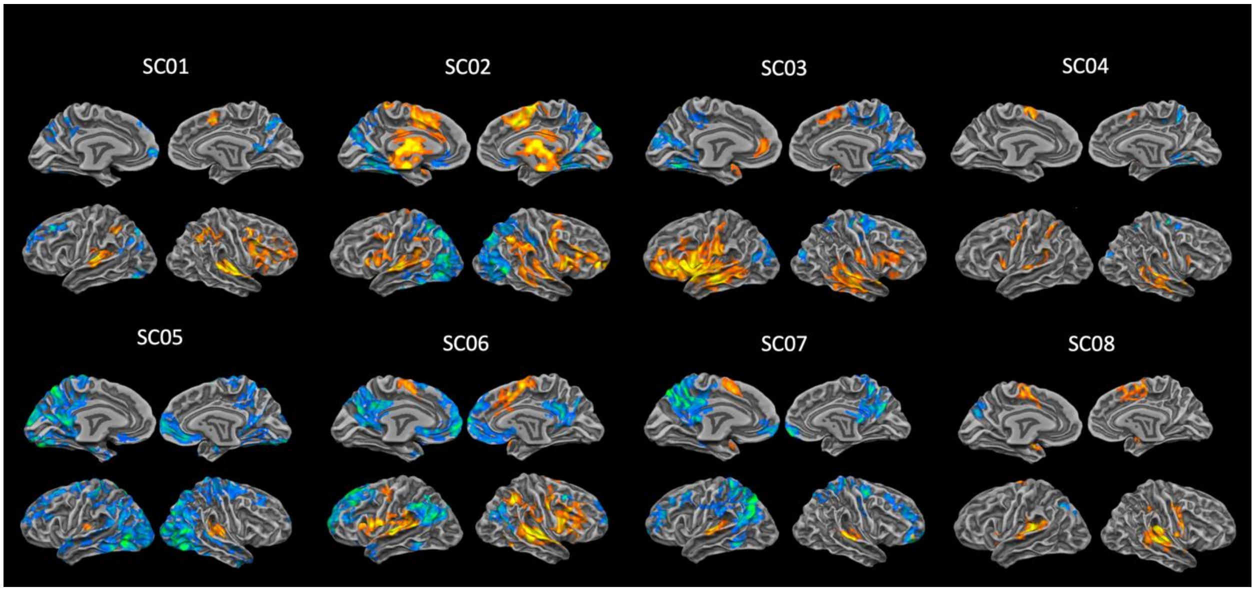

Figure S1. Sighted controls separate subject fMRI response to auditory stimulation. The auditory fMRI data were processed using the single-subject general linear model (GLM) statistics as implemented in BrainVoyager21.4. All GLM analyses were performed using FDR corrected $p<0.04$ ($q<0.05$) and an extent threshold of cca $\geq 50\ mm^2$. These results further confirm the results presented in Figure 3A and demonstrate the lack of auditory cross modal plasticity in sighted controls, except for a small area of unilateral activation of the visual cortex in SC02.

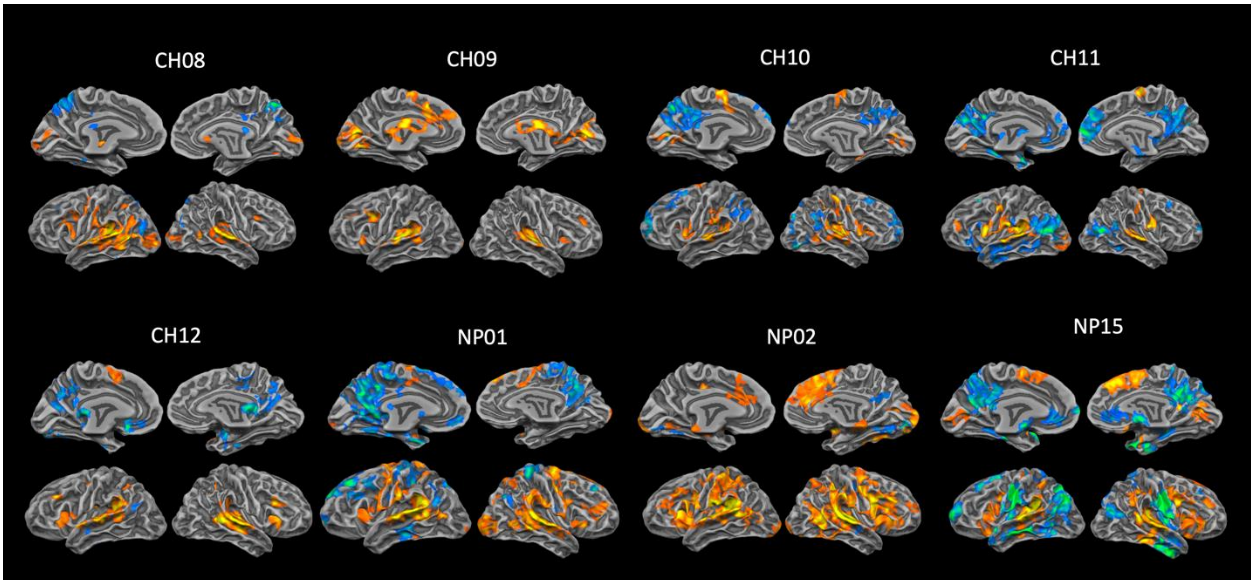

Figure S2. Auditory task fMRI results for single RPE65 patients at baseline. The single subject analyses of the RPE65 patients baseline were processed using GLM and the same statistical threshold limits of $q<0.05$ and extent threshold of ≥ 50 mm^2. Results demonstrated here show that all RPE65 patients, with the exception of CH12, present with different magnitudes of cross modal cortical activations within the primary visual cortex. CH12 was the oldest patient (44 years old) with the most advanced stage of retinal disease.

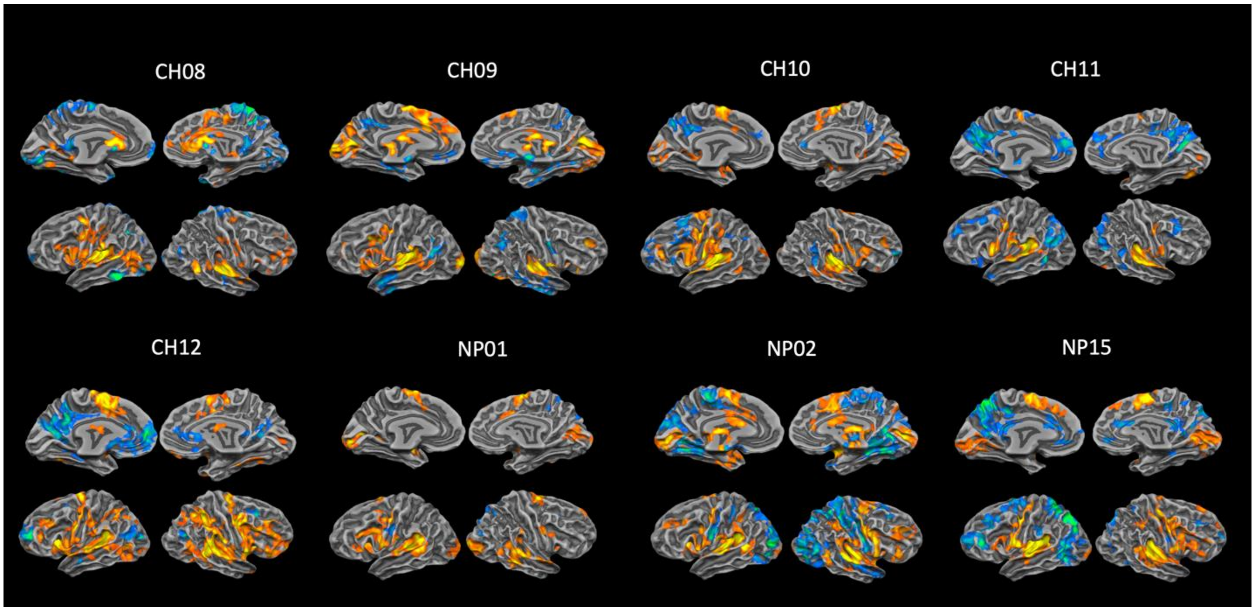

Figure S3. Auditory task fMRI results for single RPE65 patients at 3 years after retinal gene therapy. RPE65 patients auditory task fMRI data were processed using the single-subject GLM statistics at the same statistical threshold limits of $q<0.05$ and an extent threshold of $\geq$50 mm^2. All RPE65 patients, including CH12, presented with enhanced cortical activations within the primary visual cortex in conjugation with their expected cortical activations in and around the auditory areas.
